# Preparation of Hydrogen Electrodes of Solid Oxide Cells by Infiltration: Effects of the Preparation Procedure on the Resulting Microstructure

**DOI:** 10.3390/ma13010131

**Published:** 2019-12-27

**Authors:** Bartosz Hołówko, Jakub Karczewski, Sebastian Molin, Piotr Jasiński

**Affiliations:** 1 Faculty of Electronics, Telecommunications and Informatics, Gdańsk University of Technology, ul. G. Narutowicza 11/12, 80-233 Gdańsk, Poland; b.holowko@gmail.com (B.H.); pijas@eti.pg.edu.pl (P.J.); 2 Faculty of Applied Physics and Mathematics, Gdańsk University of Technology, ul. G. Narutowicza 11/12, 80-233 Gdańsk, Poland; jakkarcz@pg.edu.pl

**Keywords:** SOFC, infiltration, porosity, evaporation

## Abstract

In this work, the infiltration technique was used to produce hydrogen electrodes for solid oxide cells. Different infiltration methodologies were tested in order to try to shorten the infiltration cycle time. The porous scaffolds used for infiltration were based on highly porous yttria-stabilized zirconia (YSZ) obtained by etching the reduced nickel from the Ni-YSZ cermet in HNO_3_ acid. The support had a complex structure which included a ~130 µm porous functional layer with small pores and a ~320 µm thick supporting layer with large pores. Infiltrations have been carried out using aqueous nickel nitrate solutions. Various infiltration procedures were used, differing in temperature/time profiles. The results show that slow evaporation is crucial for obtaining a homogeneous material distribution leading to high-quality samples. A longer evaporation time promotes the proper distribution of nickel throughout the porous scaffold. The shortening of the heat treatment procedure leads to blockage of the pores and not-uniform nickel distribution.

## 1. Introduction

Nowadays, when the demand for electricity is constantly increasing, an alternative solution to currently existing energy sources is being sought. Current trends indicate the need to look for green solutions that are eco-friendly, efficient, and cheap. Fuel cells, in particular solid oxide fuel cells (SOFCs), fulfill these needs [1,2]. SOFCs convert chemical energy into electricity when working at high temperature, usually 700–1000 °C. However, this temperature should be reduced [3]. In assembly, the fuel cell should work for a very long time without the need to replace components [4]. It consists of a fuel electrode, an oxygen electrode and an electrolyte, but often it is necessary to add a diffusion layer between oxygen electrode and electrolyte for better performance [5]. Usually, the fuel in such a cell is hydrogen or hydrocarbons [2,6]. As a fuel electrode, a porous cermet made of nickel oxide as electron conductor and yttria-stabilized zirconia (YSZ) as an ionic conductor is widely used. Other materials that are nowadays developed are perovskites and double perovskites [7,8]. Porosity is important in terms of the possibility of gas penetration and increasing the triple-phase boundary—the place where electronic and ionic conductors and gas meet for electrochemical reactions occur. Cermet is a system with good catalytic and mechanical properties, especially when used together with the YSZ electrolyte. The problems of such a system are associated with the instability of nickel in the hydrogen atmosphere manifested in the agglomeration and grain growth, especially through Ostwald ripening which leads to conductivity loss [9,10,11,12,13,14]. Such percolation paths disappearance causes gradual cell degradation. Several various methods are used to produce fuel electrodes, such as tape-casting [15,16]. Recently, the unconventional method called wet infiltration has attracted much attention [17,18,19]. This method involves introducing a solution of the catalytic material dissolved in a solvent into the porous structure of the skeleton, which can be prepared by conventional method like screen printing. The most popular solvent is water with the addition of non-ionic surfactant Triton X-100 [20]. Sometimes instead of the aqueous solution, a polymeric precursor was used [21]. Thanks to this technique, it is possible to create a nanograins coating on the skeleton. Good distribution of nanostructures throughout the entire skeleton volume means that the percolation threshold of such material is lower than when using traditional methods. In addition, it increases the active surface area and catalytic activity [21,22,23]. The porosity of cermet results from the use of additional components that evaporate in the cermet formation process. The size, shape and number of pores depend strongly on pore formers. Usually, cermet porosity using common pore formers such as graphite or poly(methyl methacrylate) (PMMA) can be up to 60%, but it is uncommon. Therefore, it usually ranges from 35–40% as it is the range necessary for good performance of cell [20,24]. However, using the technique developed by Gorte et al., i.e., acid leaching the Ni-YSZ cermet, higher porosities of over 70% can be obtained. Leaching involves placing the reduced cermet in HNO_3_ acid at a temperature close to boiling. Nickel will be dissolved and YSZ will remain in the form of a skeleton since it does not interact with acid. This type of leachable pore former has the advantage of obtaining high porosity after sintering and retaining the structural features of the ionic conducting backbone [25]. Using this technique, the possibility of lowering the percolation threshold to a limit of 10% was observed [26]. Another method for lowering the amount of Ni needed is net-shape technology [27], also used on porous skeletons, but obtained without the leaching.

The infiltration process is often poorly described in the literature [18,28]. The best results seem to be obtained using capillary forces or vacuum [20,28] for impregnation in the micro-scale. The calcining process takes place at 400 °C for minimum of 10 min. This is the temperature at which evaporation of the solvent, decomposition of nitrates and formation of oxides proceed [16,23,29]. It is often performed with the previous drying or with a very slow heat rate [30]. We have found that the infiltration process has a significant influence on the properties of the fabricated composite. Whereas the infiltration method is frequently used to produce nano-featured electrodes, no specific details about the infiltration procedure are reported. Therefore, the aim of this work is to investigate the influence of the temperature-time profiles on the evaporation of the infiltrate solvent and on the microstructure of the composite. For the infiltration, a gradient porous support is used, with both the functional layer and the supporting layer, resembling the current state-of-the-art in the field of advanced cells. Due to the difference in pore sizes of the layers, it is of interest to study the infiltration of the two layers. To the best of our knowledge, no detailed studies about the impregnation of the nickel catalyst into cell gradient structures have been reported. Moreover, the porous structure for infiltration includes a 10 µm thick dense electrolyte, to reflect typical solid oxide cell structure (only one side of the cell is available for evaporation).

The results concerning the infiltration of Ni into a porous YSZ structure for electrode fabrication are relevant both for the fuel cells (SOFCs) and electrolysis cells (SOEC) development.

## 2. Experimental

The basis for infiltration was the YSZ skeleton with a high porosity obtained by HNO_3_ acid etching of nickel from a previously reduced half-cell nickel cermet from a sintered cell (delivered by the National Taipei University of Technology—NTUT, NiO/YSZ (60:40 vol.%), anode substrate composed of two layers with different degree of porosity and YSZ electrolyte made using tape casting technique). To obtain Ni-YSZ samples for leaching, the reduction step of NiO to Ni was performed at 900 °C for 10 h in 97%/3% H_2_/H_2_O. Leaching was performed in hot HNO_3_ (POCH, Gliwice, Poland) at 80 °C for about 30 min. Progress of leaching was controlled by observing sample colors and weight changes. Sample with the acid was placed in a glass beaker on a hot plate. After leaching and cleaning the sample with deionized water, the porous YSZ samples were further calcined at 1200 °C to improve their mechanical strength.

The infiltration was performed by placing the porous skeleton in a glass Petri dish and distributing the infiltration material at the periphery of the sample by micropipette in such a way that it penetrates the skeleton structure through of capillary forces. The samples were not fully soaked/immersed under the liquid, but were sucking the liquid from the sides. In addition, for assisting the infiltration, the system remained under vacuum (~30 mbar). The sample was placed in a Petri dish in such a way that the dense electrolyte was placed down with the pores facing upwards. For infiltration of the samples, a 4 M solution of nickel nitrate (Sigma Aldrich, St. Louis, MO, USA, >99.5% purity) in deionized water (DI) with 3% of Triton X-100 (Sigma Adrich, St. Louis, MO, USA) dispersant was prepared. The hot plate used was Glass Ceramic Hotplate CT 10 (Harry Gestigkeit GmbH, Dusseldorf, Germany) controlled by a surface mounted K-type thermocouple connected to Fuji PXR-4 programmable controller (Fuji, Tokyo, Japan). For Scanning Electron Microscopy (SEM) evaluation, FEI Quanta FEG 250 (FEI, Hillsboro, OR, USA) with the energy-dispersive X-ray spectroscope (EDX) EDAX Genesis APEX 2i (EDAX, Mahwah, NJ, USA), as well as Phenom XL with a dedicated EDX detector (ThermoFisher, Eindhoven, the Netherlands) were used. The X-Ray diffraction (XRD) was used to determine phase changes. The XRD measurements were performed using X’Pert Pro MPD Philips diffractometer (Philips, Eindhoven, the Netherlands) using Cu Kα (*λ* = 1.542 Å) radiation. Porosities of the samples were measured using the Archimedes principle, with kerosene as the liquid medium using a Radwag balance (Radom, Poland) with an Archimedes add-on. Kerosene is used as it is highly penetrating and has shown to provide more reproducible results compared to other liquids. For the density determination, the samples were weighed in a dry state, saturated with kerosene form, and submersed to cause a liquid displacement.

## 3. Results and Discussion

**Figure 1:**
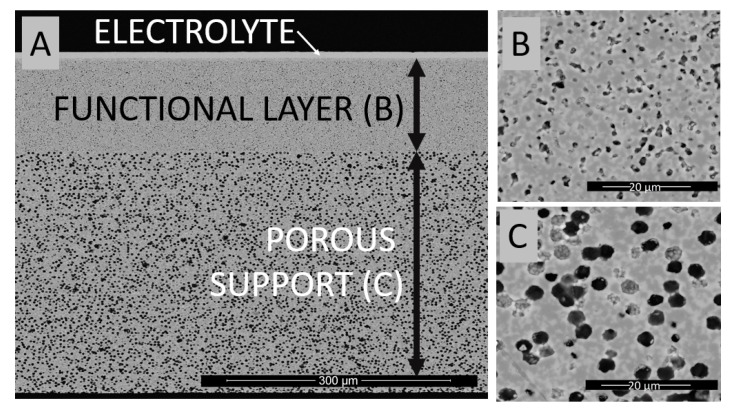
Micrograph of half-cell made using tape casting technique and consisting of two layers of NiO-yttria-stabilized zirconia (YSZ) and YSZ electrolyte: (**A**) overview of the half-cell; (**B**) magnification of the functional layer; (**C**) magnification of the porous support layer.

According to Figure 1, the initial half-cell consists of a NiO-YSZ porous parts and dense YSZ electrolyte. The electrode part is divided into two distinct layers with different microstructures. Electron microscopy imaging was used to estimate the porosity and characteristic pore size (diameter). Closer to the electrolyte, in a place where a higher triple phase boundaries are required, there is a less porous layer (~15% total porosity, average pore diameter ~1 µm). Below this layer is a more porous layer (~30% total porosity, average pore diameter ~5 µm) for improved gas distribution. The thickness of the individual layers is: electrolyte 10 µm, functional porous layer 120 µm, porous support layer 320 µm. The gradient structure of the cell represents state-of-the art on the cell construction. Due to different pore size and the presence of a dense YSZ electrolyte on top, the infiltration followed by drying and calcination forms a complex process which must be developed, described and possibly optimized for effective cell operation.

**Figure 2:**
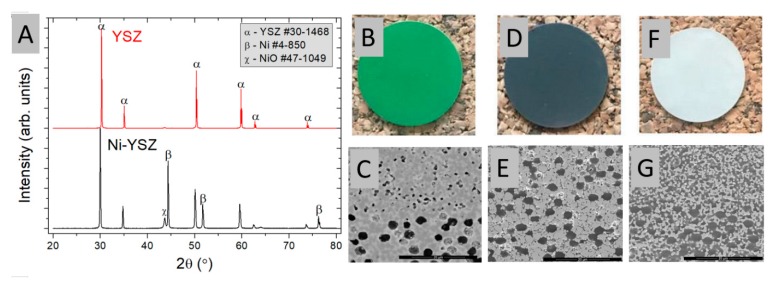
(**A**) XRD of samples after (top) and before (bottom) leaching, (**B**,**C**) optical (surface) and SEM micrograph (cross-section) of the NiO-YSZ sample before reduction and leaching, (**D**,**E**) Ni-YSZ after reduction, and (**F**,**G**) YSZ after reduction and leaching.

The half-cell shown in Figure 1 has been reduced to form Ni-YSZ and subsequently leached in hot HNO_3_ acid to remove the metal. YSZ remained unaffected by the etching. XRD and microstructural analyses performed before and after reduction and leaching, presented in Figure 2 confirm the fabrication of a highly porous YSZ sample. In addition to Ni and YSZ, a small peak corresponding to unreduced NiO phase is detected in the leached sample. Possibly some NiO was enclosed in closed porosity or not fully reduced to oxide and thus not leached. Optical analysis of the samples shows clear changes in color between the differently processed samples. Initial cell, consisting of NiO-YSZ has a green color, as expected for NiO. After reduction, the metallic/greyish color was obtained, due to presence of metallic Ni. After leaching a white-colored sample is obtained, indicating the pure YSZ phase. Based on these analyses, the small detected amount of NiO does not seem to be important for the current study.

Porosities of the samples presented in Figure 2B,D,F were measured by the Archimedes method. The overall porosities of the initial hydrogen electrode were ~23%, after reduction it was increased to ~47%, and after leaching it was ~65%. To further obtain the porosities of the individual layers, analyses of SEM images were performed. The more porous support layer reached ~35 vol.% after reduction and ~62 vol.% after leaching. The functional layer showed similar porosity after reduction (~15%, probably due to small pore size) and after leaching reached ~53 vol.%. In general, good agreement between the SEM image analysis and the Archimedes method was obtained.

**Figure 3:**
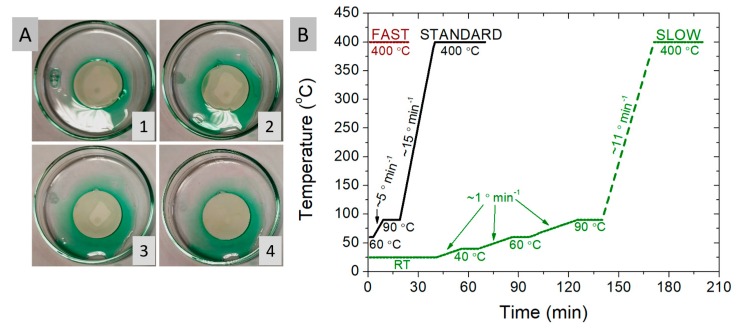
Optical images of the infiltration process of the porous YSZ skeleton (**A**, 1–4) and the hot-plate temperature heat treatment profiles evaluated in this work (**B**).

**Figure 4:**
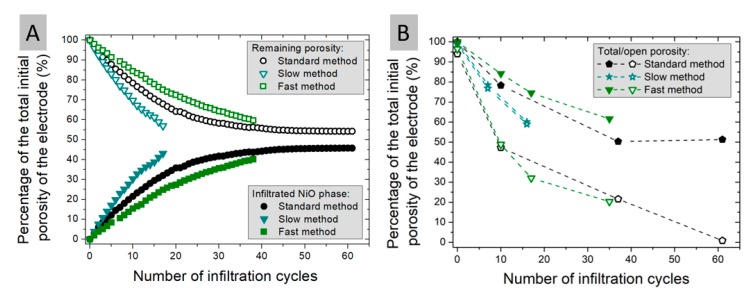
Normalized to 100% porosity curves of (**A**) changes in volume fraction of individual fractions for the sample dried by the three different methods together with (**B**) porosity obtained by Archimedes method.

For the introduction of the Ni phase by infiltration, a 4 M nickel nitrate solution with Triton-X dispersant (3 vol.%) in deionized water was used. Choosing a proper solvent system for the infiltration seems an important task. Water based solutions have a great environmental advantage over the organic based solvents, however their contact angle/wettability are not so good for highly porous samples with small pores (<5 µm). To improve wettability and infiltration, a dispersant/surfactant is added to water. Triton X-100 is a nonionic surfactant with a hydrophilic polyethylene oxide chain soluble in water, ethanol and other solvents. Based on our initial studies, adding 3 vol.% of Triton X-100 surfactant to DI water resulted in superior wettability in comparison to pure DI water and thus such a mixture was used in this work. For comparison, Briss et al. used a 9% Triton X-100 addition [15]. Optical images of the porous YSZ skeleton during the infiltration process are presented in Figure 3A. The highly porous sample (~70% initially) easily pulls the Ni-containing solution into the pores. The time-scale between pictures 1–4 is ~5 min. After achieving the saturation as presented in sub-figures 1–4 of Figure 3A, the sample is further placed in vacuum (~30 mbar) to ensure that the possible trapped air is removed and the small pores are filled with the solution.

After the infiltration, for thermal decomposition, the infiltrated material must be heated up to 400 °C. Typically, before going to 400 °C, drying steps are used to evaporate the solvent. In this paper, the goal was to evaluate methods that lead to the homogeneous distribution of nickel in the volume of the porous electrode. Three different methods, with varying the total time of a single infiltration step were considered. Usually, the infiltration procedure needs to be repeated many times to obtain a desired load of the catalyst. So the whole procedure of the preparation of the electrodes by infiltration is very time consuming and any potential decrease in the infiltration procedure time can give important gains.

In this study, we try to find the optimized conditions, shortening the infiltration steps. Therefore, the three methods are compared in this study: the “standard”, the “fast” and the “slow” methods. Their respective temperature-time profiles are presented in Figure 3A. In each case the infiltration was repeated until the maximum saturation level inside the skeleton was reached. For the “standard” method: after the infiltration of the liquid catalyst, the material was placed on a 60 °C hot plate and then heated to 90 °C for 5 min and kept at this temperature for 10 min to evaporate the water and then heated to 400 °C at a speed of 15 °C/min and held for 30 min. In the “fast” method the sample heating step was omitted. After the infiltration of the YSZ skeleton with the aqueous solution of nickel nitrate, the sample was immediately placed on a previously pre-heated hot plate held at 400 °C. The heating step of the sample was therefore very rapid. It is possible that this would limit the movement of liquids under the influence of gravity and the capillary forces acting at that time would keep the nickel particles evenly distributed throughout the skeleton. An additional advantage of this method is the shortening of the entire infiltration process to only ~30 min. During the first few infiltrations, boiling of the solution was observed and it flew out of the skeleton. Therefore, it is also possible that the reason for the formation of nickel accumulation is too fast evaporation of the solvent, which causes nitrate to be pushed to the surface. The “slow” method, for which one infiltration cycle requires ~3.5 h heat treatment (plus the infiltration stage) is by far the most time-consuming. Allowed ample time, the sample is thoroughly dried at RT, 40 °C, 60 °C, 90 °C and then slowly heated to 400 °C. Slow drying steps at 40 °C and 60 °C were used to determine the importance of slow drying/evaporation of the nickel-containing solution. Faster drying/evaporation is believed to pull the dissolved nickel in the liquid phase, whereas slow evaporation immobilizes nickel where the precursor has dried.

The highest temperature in the heat treatment profile (~400 °C) is based on the thermal decomposition profile of Ni(II) nitrate hexahydrate. Based on results obtained by Elmasry et al. [31], the final decomposition product of nickel nitrate hydrate is the NiO, obtained at 350 °C. Several intermediate steps exist in the decomposition profile: dehydration and removal of NO_x_, so that the intermediate phases (e.g., Ni(OH)_2_) are formed.

For microstructural evaluation, it was decided to produce samples with all three methods. The goal was to introduce ~30–35 vol.% (bulk volume of the skeleton) of NiO into the skeleton by multiple infiltration steps. This amount of NiO corresponds to ~20–25 vol.% of Ni after reduction. That, in turn, is equal to the amount of the YSZ phase in the skeleton, thus it is more than enough to ensure electrode conductivity and performance (the typical composition of the hydrogen electrode is ~50:50 vol.% of Ni:YSZ). For each infiltration cycle, samples were weighed to make a curve of changes in volume fraction in the system. In addition, several measurements of porosity were performed using the Archimedes method at various theoretical levels of saturation to confirm compliance with the theoretical calculations. The results of the calculated porosity changes are presented in Figure 4A,B. The plot takes into consideration only change in the porosity, i.e., the porosity of ~70% obtained after leaching of the initial Ni from the Ni-YSZ skeleton is rescaled to 100%, and only the filling of the porosity is taken into account. After infiltration of the ~30 vol.% NiO, the porosity of the skeleton still remains at the level of ~50%, which would further increase after the reduction of NiO to Ni. The remaining porosity value is calculated taking into account the volume of the infiltrated phase. The plot of the remaining porosity shown in Figure 4A can be compared with measured porosity data presented in Figure 4B. Deviations between the theoretically predicted porosities and the measured ones can point to possible pore-clogging and formation of closed porosity.

As presented in Figure 4A,B, different infiltration procedures lead to different filling of the porous skeleton. The “slow” infiltration method leads to larger filling of the pores (considering equal infiltration cycles, e.g., 10). For 10 infiltration cycles, the “slow” method filled roughly ~30% of the available porosity, the “normal” method ~20% and the fast method only ~13% of the available space. Porosity measurements (Figure 4B) carried out on the samples at different stages of infiltration show interesting features. For the samples obtained via the “fast” and the “standard” methods, a large decrease of the measured porosity is visible, not compatible with the porosity calculated from the weight change (Figure 4A). Also, visible is the discrepancy between the total and open porosities measured for these samples. This might be connected to the partial closing of the sample porosity by the formation of an outer shell or some internal blocking of pores by too fast evaporation etc. This will be evidenced later on in the paper. For the sample obtained via the slow process, the profiles of the remaining porosity and the measured total/open porosity from Figure 4A,B overlap. For the slow processed sample, the total porosity is equal to open porosity. This suggests homogeneous filling of the porous matrix with no blocking of the porosity.

An important conclusion can be drawn here: although the slower infiltration cycle is much longer than the standard cycle (~3.5 h vs. ~1.25 h), the number of infiltration cycles to obtain the same loading of the catalyst is lower, i.e., to obtain 30 vol.% NiO in the skeleton, 10 slow cycles are required, whereas for the fast method the number of cycles is ~25. Therefore, in terms of the total time of the procedure, there is no real improvement in the processing time due to use of the fast infiltration method.

**Figure 5:**
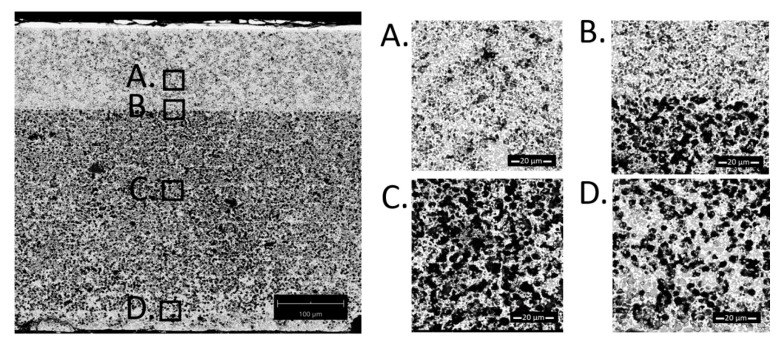
SEM cross-section images of a sample with 34 vol.% NiO obtained using the “standard” method. (**A**) is the region of the functional layer, (**B**) is the interface between the functional and the porous layer, (**C**) is the porous functional layer and (**D**) is close to the outer edge of the cell.

In order to determine the appearance of the microstructure of the fabricated substrate, an SEM test was performed. The obtained micrographs of the sample made using the “standard” method are shown in Figure 5. The sample had ~34 vol.% of the infiltrated NiO obtained in 20 infiltration cycles, which corresponds to ~26% Ni content after reduction.

The microstructure is characterized by an uneven dispersion of the nickel oxide phase, forming barriers at the skeleton edge (Figure 5D), causing no further penetration of the infiltration solution into the sample at a certain infiltration level. The material was reduced at 900 °C for 10 h in an atmosphere of humidified hydrogen (97% H_2_/3% H_2_O). After this procedure, the expected increase in porosity due to oxygen loss was observed. This structure explains the increase in open porosity reaching the level of the total porosity (as presented in Figure 4A,B). The saturation in the oxidized state results from closing the paths for penetration of the liquid solution.

**Figure 6:**
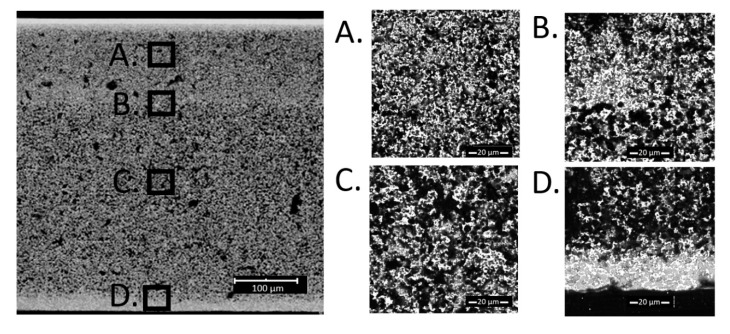
SEM cross-section images of a sample with 30 vol.% NiO obtained using the “fast” method. (**A**) is the region of the functional layer, (**B**) is the interface between the functional and the porous layer, (**C**) is the porous functional layer and (**D**) is close to the outer edge of the cell.

SEM test for the sample made using the “fast” method was carried out. The results are shown in Figure 6. The sample had ~30 vol.% of the infiltrated NiO obtained in 25 infiltration cycles, which corresponds to ~22% Ni content after reduction.

From the obtained micrographs it can be concluded that the proposed method does not result in better distribution of the infiltrated material compared to the standard method. Two areas of nickel oxide accumulation are visible, especially as seen in part D in Figure 6. In addition, some accumulation occurred at the junction of cermet with YSZ electrolyte (Figure 6B) and at the edges of the cermet, blocking liquid access from the sides. Due to the results of previous experiments, it was found that the likely reason for the formation of nickel accumulation is too fast evaporation of the solvent, which causes nitrate to be pushed to the surface.

**Figure 7:**
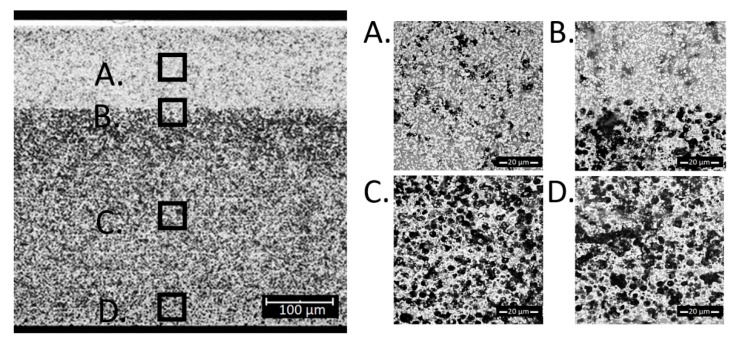
SEM cross-section images of a sample with 36 vol.% NiO obtained using the “slow” method. (**A**) is the region of the functional layer, (**B**) is the interface between the functional and the porous layer, (**C**) is the porous functional layer and (**D**) is close to the outer edge of the cell.

To determine the microstructure obtained using the “slow” method, SEM tests were performed. The results are shown in Figure 7. The sample had ~36 vol.% of the infiltrated NiO obtained in 16 infiltration cycles, which corresponds to ~27% Ni content after reduction.

No barrier or larger nickel oxide accumulation sites were observed in the images. A large part of nickel is accumulated in the functional layer (part A in Figure 7), which will have a positive effect during cell operation. Due to smaller porosity and larger capillary forces, the filling of the functional layer is more efficient than the filling of the more porous supporting layer. Overall, the resulting microstructure indicates the correct dispersion of nickel oxide in the entire volume of the skeleton.

Based on Figure 5, Figure 6 and Figure 7, porosities of the individual layers were determined by SEM analyses. The data is summarized in Table 1. For the sample obtained by the “fast” method, the porosity of the functional layer does not differ from the initial sample. As seen in Figure 6, most Ni accumulated in the blocking layer. Analyzing the data obtained for the samples prepared by the standard and the slow methods, it seems that the functional layer, with smaller pore size, fills preferentially with nickel.

**Figure 8:**
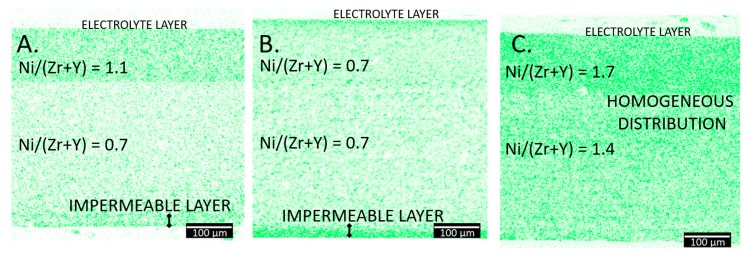
Nickel elemental maps (based on EDX analyses) of samples with (**A**) 34 vol.% NiO obtained using the “standard” method, (**B**) 30 vol.% NiO obtained using the “fast” method (**C**). 36 vol.% NiO obtained using the “slow” method. Dense electrolyte is in the top part of the images, followed by the functional layer and the porous support.

In order to further compare the microstructures of the produced cells, additional EDX tests were performed to illustrate the distribution of nickel oxide in the individual methods used in this work. The obtained images in the form of Ni elemental maps throughout the entire cross-section shown in Figure 8.

The obtained EDX maps show the differences in nickel distribution over the entire volume of the electrode substrate depending on the infiltration method used.

The Ni map images for samples prepared using the “standard” method (Figure 8A) and the “fast” method (Figure 8B) show nickel accumulation in certain areas on the outer edge (denoted as the impermeable layer) and near the electrolyte. This creates a barrier through which an aqueous solution of nickel nitrate cannot further pass, which hinders or completely prevents further penetration of the skeleton. Only for the sample prepared by the “slow” (Figure 8C) method, a significant saturation and uniformity of the nickel phase is observed in the entire volume of the skeleton.

In order to quantify the amount of the infiltrated nickel, atomic compositions were determined based on the EDX analyses results. Results, given as the Ni/(Zr + Y) ratios, are included in Figure 8. The relative amount of Ni differs between the samples. Even though the samples contain a similar Ni amount, its distribution is different. Accumulation of the nickel at the edge (as the impermeable layer) decreases the effective Ni content in the support and functional electrode parts. The relative amount of Ni in the “slow” infiltrated sample is much higher than the amount in the two other samples. Results presented in Figure 8 confirm, that more nickel accumulates in the functional layer with the small pores than in the porous layer. This effect should be beneficial for the electrochemical performance of the cell.

Based on the developed methodology, fabricated substrates will be studied for their electrical and electrochemical properties in the near future.

## 4. Conclusions

Infiltration is one of the most promising methods to obtain efficient hydrogen electrodes for intermediate temperature fuel cells. The current state of the art hydrogen electrodes is formed by advanced gradient structures with different pore sizes, which alter the infiltration procedure/results. Unfortunately, the details about the infiltration procedures are often overlooked in the literature. This work focused on the evaluation of three different procedures for infiltration, differing in total time required for one infiltration cycle. A complex bi-layer, highly porous YSZ skeleton (~70% porosity) was used as the substrate. It has been obtained by leaching of the Ni from the Ni-YSZ cermet. The microstructure of the sample infiltrated with the “slow” method (~3.5 h per one infiltration cycle) shows homogeneous nickel distribution in the entire support volume. The amount of infiltrated material with each cycle is the least variable for this method. An even distribution of nickel oxide is observed throughout the entire sample volume, and there are no barriers causing the blocking of the skeleton to penetrate the material with the aqueous solvent. Therefore, considering the microstructure, the material prepared in this way meets the conditions of the cermet as an electrode substrate for solid oxide cells. The conclusion is that by using the slowest evaporation, no changes are made to the nickel compounds and are not forced to move due to the evaporation of the solvent. As a result, thanks to capillary forces, it is possible to maintain the desired infiltrated compounds.

## Figures and Tables

**Table 1:** Porosity values determined for the studied samples (based on SEM images analysis).

Sample	Infiltrated Phase [vol.%]		Porosity [vol.%]	
	NiO: (Measured)	After Reduction: (Estimated)	Functional Layer	Support Layer
Initial (leached)	-	-	~52	~62
The fast	~30	~22	~59	~45
The standard	~34	~26	~30	~50
The slow	~36	~27	~24	~50
